# Pattern Recognition of Partial Discharge Faults in Switchgear Using a Back Propagation Neural Network Optimized by an Improved Mantis Search Algorithm

**DOI:** 10.3390/s24103174

**Published:** 2024-05-16

**Authors:** Zhangjun Fei, Yiying Li, Shiyou Yang

**Affiliations:** College of Electrical Engineering, Zhejiang University, Hangzhou 310027, China; feizj2013@163.com (Z.F.); eesyyang@zju.edu.cn (S.Y.)

**Keywords:** back propagation neural network, mantis search algorithm, partial discharge pattern recognition, principal component analysis, switchgear

## Abstract

The dependable functioning of switchgear is essential to maintain the stability of power supply systems. Partial discharge (PD) is a critical phenomenon affecting the insulation of switchgear, potentially leading to equipment failure and accidents. PDs are generally grouped into metal particle discharge, suspended discharge, and creeping discharge. Different types of PDs are closely related to the severity of a PD. Partial discharge pattern recognition (PDPR) plays a vital role in the early detection of insulation defects. In this regard, a Back Propagation Neural Network (BPNN) for PDPR in switchgear is proposed in this paper. To eliminate the sensitivity to initial values of BPNN parameters and to enhance the generalized ability of the proposed BPRN, an improved Mantis Search Algorithm (MSA) is proposed to optimize the BPNN. The improved MSA employs some boundary handling strategies and adaptive parameters to enhance the algorithm’s efficiency in optimizing the network parameters of BPNN. Principal Component Analysis (PCA) is introduced to reduce the dimensionality of the feature space to achieve significant time saving in comparable recognition accuracy. The initially extracted 14 feature values are reduced to 7, reducing the BPNN parameter count from 183 with 14 features to 113 with 7 features. Finally, numerical results are presented and compared with Decision Tree (DT), k-Nearest Neighbor classifiers (KNN), and Support Vector Machine (SVM). The proposed method in this paper exhibits the highest recognition accuracy in metal particle discharge and suspended discharge.

## 1. Introduction

Switchgear is an essential infrastructure in urban power distribution networks. Consequently, the reliability of a power supply is directly related to that of a switchgear. However, a switchgear faces various issues, such as contamination effects, weak insulations, inadequate creepages and air clearances, insufficient manufacturing and assembly qualities, inadequate contact capacities, or poor contacts, in design, manufacturing, installation, and maintenance, resulting in a higher accident rate. Condition-based maintenance is a promising technical means to improve the reliability of power supply equipment. However, due to the large number of high-voltage switchgears and the low equipment cost of a switchgear as compared to the high cost of a monitoring device, it is infeasible to use an online detection approach such as high-voltage transformers for a switchgear to achieve comprehensive and real-time online monitoring.

The dielectrics or insulator in a switchgear is exposed to an electric field in operation. Once the electric field strength in a certain area exceeds the breakdown threshold of the material, discharging will occur in that area in question. Nevertheless, this discharging will not penetrate the entire insulation system between two conductors under the applied voltage. This phenomenon is called the partial discharge (PD). PD can lead to a degradation of the insulation and the vicious cycle of defects in the power equipment; in severe cases, it can even cause insulation accidents. Therefore, PD detection is essential in preventing sudden insulation damage accidents of a power equipment. The partial discharge faults in a switchgear are generally grouped into metal particle discharge, suspended discharge, and creeping discharge. Different types of PD are closely related to the severity of a PD. The partial discharge pattern recognition (PDPR) is thus used to accurately identify and categorize the PD events based on their unique patterns. The output information of PDPR is valuable for condition monitoring and the maintenance of the electrical equipment, as it allows for the early detection of insulation defects and the implementation of appropriate corrective actions to prevent equipment failure and ensure the reliability and safety of the power electrical system. Consequently, the PDPR is of great significance to the reliable operation of a switchgear. 

The PDPRs are typically based on the signal characteristics generated in PD events, which can be classified into two major categories: chemical signals and physical signals. Chemical signals are mainly various gas derivatives produced in PD [1,2,3,4]. Muhamad et al., employed the random forest (RF) algorithm to conduct the pattern recognition of a gas insulated switchgear (GIS) using 12 by-product gases, and the performance of RF confirmed the feasibility of eight algorithms [3]. Rao et al., proposed a feature selection and ensemble learning based methodology to diagnose a transformer fault based on dissolved gas analysis data, and the proposed methodology achieved a 100% accuracy in recognizing fault types of the IEC TC 10 database [4]. Physical signals typically encompass heat, electromagnetic, and acoustic signals detected in PDs. Polužanski et al., investigated the influence of the power transformer oil-temperature on the accuracy for PD locations [5]. Lu et al., proposed a defect recognition method for GIS PD based on YOLOv5 and ultra-high frequency (UHF) sensor-detected electromagnetic signals. [6]. He et al., utilized a combination of acoustic and electrical signals for GIS PD localization [7].

In view of the detected PD electrical or transformed electrical signals, the characteristics, such as the amplitude, the frequency, the duration, and the waveform shape, are first analyzed to extract the relevant features, and the relevant features are then used to trained classifiers to recognize and classify the types of PD patterns of a PD. These classifiers can be based on machine learning algorithms like artificial neural networks (ANNs) [8,9], Support Vector Machines (SVM) [10,11], fuzzy logic control [12], or random forest (RF) [13]. The design of the classifier is a crucial part of PDPR. Zhu used an ANN identification method to identify UHF PD patterns in electrical equipment [8]. Li et al., used the least squares SVM (LS-SVM) to analyze the PD development stages of a converter transformer based on the PD statistical characteristics of the phase-resolved partial discharge spectrogram, equivalent time-frequency spectrogram, and discharge pulse waveform [11]. To address the issue of insufficient partial discharge data and the low generalization capability caused by the imbalanced data in field conditions, Zhu et al., proposed an improved WGAN-GP model that generates data samples of different classes conditionally while ensuring the training process stability [14]. This model was used for enhancing PD data in power equipment and detecting PD signals. Xi et al., introduced an attention mechanism (AM) into the PDPR model to improve the recognition accuracy and the computational complexity by emphasizing its effective characteristics and combining the past and future information [15]. They proposed an intelligent partial discharge detection model based on the Bi-directional Long Short-Term Memory (AM-Bi-LSTM) network for identifying partial discharge faults in Insulated Overhead Conductors (IOC)s. Rizzi et al., used a genetic algorithm to extract key features and selected neuro-fuzzy classifiers for PDPR of cable partial discharge [16]. Feng et al., started from the phase resolved partial discharge spectrogram, extracted the moment features and gray level co-occurrence matrix features of the spectrogram, and used a SVM to classify the combined 7-dimensional features [17]. Hao et al., used a logistic pattern tree that combines decision trees and logistic regressions to identify partial discharges in switchgears [18]. Jing et al., applied K-Means clustering for pattern recognition of detected partial discharge signals in oil-paper insulated cables based on polar coordinate spectra, even in the absence of phase information [19]. Lv et al., utilized a particle swarm optimization algorithm to optimize a multiple kernel learning relevance vector machine model for GIS discharge pattern recognitions [20]. Yao et al., extracted features from the PD spectrogram using a three-parameter Weibull model and employed the Grey Wolf Optimization algorithm for fast and accurate determinations of the Weibull model parameters [21]. The obtained model effectively represents the characteristics of the PD occurrence and discharge magnitude distribution spectrogram. Wei et al., used a simulated annealing algorithm and an ant colony algorithm to optimize four different classifiers: decision tree, RF, gradient boosting and SVM, and performed pattern recognitions on ultrasonic inspection data of oil-paper insulations [22]. The results show that the classifier optimized by intelligent algorithms can further improve the recognition accuracy. Li et al., utilized a SVM model based on the differential evolution adaptive bacterial foraging optimization (DEABFO) algorithm to fulfill the GIS PD diagnosis [23]. Phase-resolved pulse sequence signals were used to extract Zernike moment features, and the results proved the effectiveness of the proposed method in PD pattern recognition applications. 

Furthermore, methods based on Convolutional Neural Network (CNN) have demonstrated their advantages in the identifications of PD. Sun et al., proposed a CNN based classifier for high-speed electric multiple units [24]. The results demonstrated that the proposed classifier outperformed the two existing NN-based classifiers in accurately identifying signals from four defect types. Jing et al., proposed an improved Generative Adversarial Network (GAN) with an auxiliary classifier to address the challenges of small and unbalanced sample pattern recognition in gas insulated switchgear PD [25]. Aldosari et al., combined the LSTM networks and the CNN to detect the PD patterns, demonstrating that the integrated CNN-LSTM network outperformed standalone CNN or LSTM networks [26]. Yin et al., developed a model to identify the statistical parameters of PD using the Hausdorff-like distances [27]. They also employed an enhanced CNN for PD pattern recognition, then employed the Dempster-Shafer (D–S) evidence theory to merge the outcomes of the two pattern recognition methods, thereby enhancing the accuracy of PD pattern recognition. However, owing to the intricate nature of image processing and the substantial convolution operations and parameters involved, CNNs often entail a higher parameter count and require more computational resources compared to traditional machine learning methods. 

A Back Propagation Neural Network (BPNN) is a multi-layer feedforward network with a strong nonlinear approximation capability. Existing researches have demonstrated the effectiveness of BPNN in PDPR [13,28]. Since PD has obvious randomness, and the degree of a discharge is closely related to factors such as the location where the discharge occurs, the local field strength of the discharge, and the applied time instant of a voltage, an identification method oriented to random features should be used, that is, extracting statistical parameter features from PDs. The signal feature quantity determines the appropriate feature space for the PDPR system and provides a good foundation for a classifier design. However, the existing BPNN classification method for PDPR has a certain degree of subjectivity in the selection of feature parameters. It usually relies on an expert experience, has serious information losses, and lacks a certain degree of generalizations, resulting in a low recognition rate [28]. In addition, some studies usually use human trial and error experiments in the selection of statistical feature quantities. However, this requires a large amount of calculations and cannot guarantee that all possibilities are traversed. Moreover, the back propagation (BP) neural network is sensitive to the initial values of network parameters, and the algorithm requires a long training time and is prone to falling into a local minimum [29]. Nevertheless, it is demonstrated that an optimized classifier by an intelligent optimization algorithm will significantly improve its classification performances [10].

In this regard, this article proposes an improved Mantis Search Algorithm (MSA) [30] to optimize BPNN to deal with the shortcomings of the BPNN being sensitive to the initial values of network parameters and uses the optimized BPNN for PDPR in a switchgear. The PDPR procedure is as follows: firstly, the ultrasonic detection approach is used to obtain partial discharge spectrograms under different patterns; then, statistical feature parameters are extracted from the phase resolved partial discharge (PRPD) spectrograms obtained from the ultrasonic detection; next, Principal Component Analysis (PCA) is used to reduce the dimensionality of the statistical feature parameters. The processed features are then input into the BPNN for learning, with four-fifths of the data used as training data and one-fifth as validation and testing data. The improved MSA algorithm is used to optimize the initial values of the neural network parameters. Finally, the trained BPNN, optimized by the improved MSA, is used for the recognition of partial discharge patterns. The contribution of this paper not only addresses a specific limitation of the BPNN, but also offers a novel approach to improving the performance and robustness of neural network optimization, thereby potentially enhancing the applicability of BPNN in various domains.

The rest of the paper is structured as follows. Section 2 presents the acquisition of initial PD signal data, the extraction of statistical features from the initial signals, and the PCA of the extracted features. Section 3 reviews the MSA algorithm and describes the improvements introduced in this paper. Section 4 employs the proposed improved MSA algorithm for PDPR and discusses the recognition results with other different methods. Section 5 concludes the work of this paper.

## 2. Experimental Data Acquisition and Statistical Feature Extraction 

### 2.1. Switchgear Partial Discharge Data Acquisition

**Figure 1:**
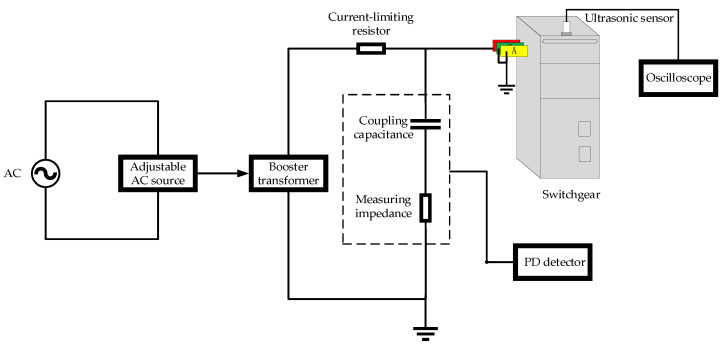
The test device of partial discharge detection for switchgear.

**Figure 2:**
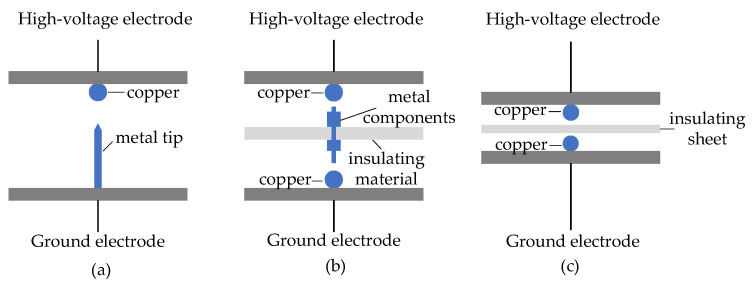
Schematics of three types of PD occurring prototypes, (**a**) metal particle discharge, (**b**) suspended discharge, (**c**) creeping discharge.

This paper developed a PD defect simulation test device for the switchgear. The components of the prototype include busbars, a circuit breaker, a current transformer, a voltage transformer, an arrester, and an insulator. The test voltage is obtained from the non-partial discharge booster transformer. A high-precision PD detector is used to detect the apparent discharge of partial discharge under simulated defects. The PD spectrogram and amplitude of partial discharge ultrasonic detection signals under simulated defects are obtained by using an ultrasonic sensor. The structure of the test device is shown in Figure 1. In Figure 1, the resistance-capacitance voltage dividing device is composed of coupling capacitance and measuring impedance, and the high-precision PD detector constitutes the PD pulse current test circuit. The ultrasonic sensor installed on the top gap of the switchgear has the function of testing ultrasonic wave spectrogram. The schematic diagrams of three types of PD occurring prototypes are shown in Figure 2. The metal particle discharge occurring prototype is composed of a copper ball electrode and a metal tip. The suspended discharge occurring prototype involves the use of an insulating sheet to secure a metal component between two copper ball electrodes. the surface discharge occurring prototype is achieved by placing an insulating sheet between the two copper ball electrodes.

### 2.2. Extraction of Statistical Features

Before extracting statistical features, the measured PD signal data need to be analyzed. In this regard, the PRPD spectrogram, describing the correspondence between the charge magnitude q and the phase φ (0°~360°), denoted by H(q,φ), of a discharge, is used. In the following description, $H_{q\max}(\varphi )$ and $H_{q\mathrm{mean}}(\varphi )$ are, respectively, the maximum magnitude, and the averaged magnitude of the charges in phase φ, for a series discharging tests. The voltage pulse magnitude is used for the discharge charge magnitude in ultrasonic detections since the latter cannot be calibrated directly. 

**Figure 3:**
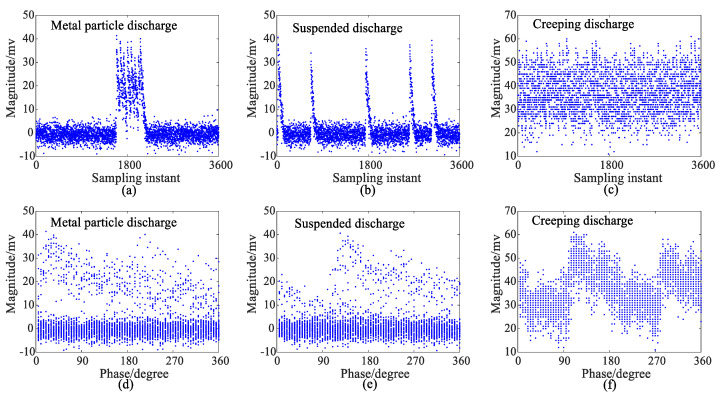
PRPD spectrogram of metal particle discharge, suspended discharge, and creeping discharge ultrasonic signals, (**a**−**c**): Magnitude versus sampling instant, (**d**−**f**) Magnitude versus phase.

In this paper, the data of discharging for a total of 50 power frequency cycles at every 5° degree, called a phase window, obtained by an ultrasonic detection are used. The recorded PRPD spectrogram distributions of metal particle discharge, suspended discharge, and creeping discharge signals are shown in Figure 3.

From Figure 3, it can be observed that the PRPD spectrograms exhibit significant differences in shapes and magnitudes for different types of partial discharge patterns. The data obtained directly from images or waveforms are quite extensive, making it challenging to perform a direct PDPR. Therefore, it is necessary to extract features from the raw data. Currently, the existing statistical features used to describe PRPD spectrograms primarily include the skewness, the kurtosis, the local peak count [31], which describe the shape differences in the spectrogram, as well as the cross-correlation coefficient, which describes the differences in the positive and negative half-cycle profiles of the spectrogram [32].

Skewness, Sk, reflects the left and right skewness of the spectrogram shape relative to the normal distribution, and it is determined from the following:

(1) $$Sk=\frac{\sum_{i=1}^{W}{\left(x_{i}-\mu \right)}^{3}p_{i}\Delta x}{\sigma^{3}}$$

where W is the number of phase windows in a half cycle; $x_{i}$ is the phase median of the *i*th phase window; Δx is the width of the phase window; $y_{i}$ is the discharge charge magnitude; $p_{i}$ is the discharge probability, defined by $p_{i}=y_{i}/\sum_{i=1}^{W}y_{i}$; μ and σ are the mean and the standard deviation when the PRPD spectrogram is regarded as a probability density distribution, defined by $\mu =\sum_{i=1}^{W}(x_{i}\cdot p_{i})$ and $\sigma^{2}=\sum_{i=1}^{W}[{\left(x_{i}-\mu \right)}^{2}\cdot p_{i}]$, respectively. 

From Equation (1), it is obvious that, the final statistical parameters for skewness include the positive half−cycle skewness $Sk_{\max}^{+}$ of $H_{u\max}(\varphi )$, the negative half-cycle skewness $Sk_{\max}^{-}$ of $H_{u\max}(\varphi )$, the positive half-cycle skewness $Sk_{mean}^{+}$ of $H_{u\mathrm{mean}}(\varphi )$, and the negative half-cycle skewness $Sk_{mean}^{-}$ of $H_{u\mathrm{mean}}(\varphi )$. Moreover, Sk=0 means that the spectrogram shape is symmetrical; Sk>0 means that the spectrogram shape is skewed to the left relative to the normal distribution shape; Sk<0 means that the spectrogram shape is skewed to the right.

Kurtosis, Ku, describes the degree of the prominence of the shape distribution compared to the shape of a normal distribution, and is calculated by using the following:

(2) $$Ku=\frac{\sum_{i=1}^{W}\left[{\left(x_{i}-\mu \right)}^{4}\cdot p_{i}\Delta x\right]}{\sigma^{4}}-3$$

Therefore, the final statistical kurtosis statistical parameters include the positive half-cycle kurtosis $Ku_{\max}^{+}$ of $H_{u\max}(\varphi )$, the negative half-cycle kurtosis $Ku_{\max}^{-}$ of $H_{u\max}(\varphi )$, the positive half-cycle kurtosis $Ku_{mean}^{+}$ of $H_{u\mathrm{mean}}(\varphi )$, and the negative half-cycle kurtosis $Ku_{mean}^{-}$ of $H_{u\mathrm{mean}}(\varphi )$. Moreover, Ku=0 indicates that the spectrogram profile is a standard normal distribution; Ku>0 indicates that the spectrogram profile is sharper and steeper than the normal distribution profile, and Ku<0 indicates that the spectrogram profile is flatter than the normal distribution profile.

The local peak, Pe, is the number of local peaks of a spectrogram. The point $\left(\varphi_{i},y_{i}\right)$ is a local peak point if the following condition is valid at point $\left(\varphi_{i},y_{i}\right)$:

(3) $$\begin{array}{ll} & \frac{dy_{i-1}}{d\varphi_{i-1}}>0\;\&\;\frac{dy_{i+1}}{d\varphi_{i+1}}<0\\ \Rightarrow & \frac{y_{i}-y_{i-1}}{\varphi_{i}-\varphi_{i-1}}>0\;\&\;\frac{y_{i+1}-y_{i}}{\varphi_{i+1}-\varphi_{i}}<0 \end{array}$$

Therefore, the final statistical parameter of the local peak is the summation of the number of the local peak points $Pe_{\max}^{+}$ in the positive half cycle of $H_{u\max}(\varphi )$, the number of the local peak points $Pe_{\max}^{-}$ in the negative half cycle of $H_{u\max}(\varphi )$, the number of local peak points $Pe_{mean}^{+}$ in the positive half cycle of $H_{u\mathrm{mean}}(\varphi )$, and the number of local peak points $Pe_{mean}^{-}$ in the negative half cycle of $H_{u\mathrm{mean}}(\varphi )$.

The cross-correlation coefficient, cc, represents the similarity of the shape between the positive and the negative half-cycles of a spectrogram. cc value close to 1 means that the profiles of the positive and negative half cycles of the spectrogram are similar in identity, and cc close to 0 indicates a significant difference in the contour of the spectrogram. cc is given by the following:

(4) $$cc=\frac{\sum_{i=1}^{W}(y_{i}^{+}y_{i}^{-})-\frac{\sum_{i=1}^{W}y_{i}^{+}\sum_{i=1}^{W}y_{i}^{-}}{W}}{\sqrt{\frac{\sum_{i=1}^{W}{\left(y_{i}^{+}\right)}^{2}-(\sum_{i=1}^{W}y_{i}^{+}{)}^{2}}{W}\cdot \frac{\sum_{i=1}^{W}{\left(y_{i}^{-}\right)}^{2}-(\sum_{i=1}^{W}y_{i}^{-}{)}^{2}}{W}}}$$

where $y_{i}^{+}$ and $y_{i}^{-}$ are the discharge voltage pulse values within the positive and negative half-cycle phase windows i, respectively. Therefore, the final statistical cross-correlation coefficient statistical parameters include the cross-correlation coefficient $cc_{\max}$ of $H_{u\max}(\varphi )$ and the cross-correlation coefficient $cc_{mean}$ of $H_{u\mathrm{mean}}(\varphi )$.

Using $H_{u\max}(\varphi )$, $H_{u\mathrm{mean}}(\varphi )$, a total of 14 statistical parameters, $x_{1}-x_{14}$, for the 3600 measured data of discharging are extracted and tabulated in Table 1. To eliminate the influence of different units of the measurement for various statistical parameters, all statistical parameters are subjected to a standardization.

### 2.3. Principal Component Analysis

If the extracted 14 statistical feature parameters are directly used as the input data of the neural network for PDPR, the dimensionality is extremely large, which is not conducive to network training. Moreover, the structure of the BPNN will be extremely complicated if too many statistical features are used, resulting in a more difficult and time-consuming training process. On the other hand, different statistical parameters may carry overlapping statistical information, and there may be certain correlations between the variables. The existing approach to dress this issue is to select a combination of different features based on an expert experience or artificial selection based on the minimum error rate of the classifier. However, such an approach cannot quickly, accurately, and comprehensively reflect the internal relationship of the statistical parameters. To address the deficiency of the existing approaches, this paper uses PCA on the 14 statistical feature parameters to extract fewer comprehensive indicators to represent various types of information existing in each variable.

PCA is used for analyzing a multivariate statistical distribution of data to reduce the dimensionality of the data. The main idea of PCA is to map the original n dimensional features space onto a new k dimensional orthogonal feature space. The k new dimensions are also known as the principal components of the n dimensional ones. The PCA algorithm is implemented based on an eigenvalue decomposition covariance matrix by sequentially finding a set of mutually orthogonal coordinate axes from the original space. The first new coordinate axis is selected to be the direction with the largest variance in the original data, and the second new coordinate axis is selected to be the plane that is orthogonal to the first coordinate axis, and the third one is the plane that is orthogonal to the 1st and 2nd axes. By repeating this procedure, one can get *k* coordinate axes. Moreover, most of the variance is contained in the first k coordinate axis, and the variance contained in the subsequent coordinate axes is almost 0. Therefore, one can ignore the remaining coordinate axes and only keep the first k coordinate axis that contain the vast majority of the variance. In fact, this is equivalent to preserving the dimensions of the features that contain the majority of the variance and disregarding the dimensions with almost zero variance, achieving a dimensionality reduction in the data features [33].

The aforementioned PCA is performed on the 14 extracted statistical feature parameters of the three different PD patterns. The sample sizes for metal particle discharge, suspended discharge, and creeping discharge are 485, 397, and 61, respectively. The number of principal components is determined based on the number of the principal components corresponding to eigenvalues that achieved a cumulative contribution rate of 95%. To give an intuitive image of the relationship between the original features and their principal components, the principal coefficients, as defined in [33], for the suspended discharge of this case are given in Table 2, a 95% cumulative contribution rate of eigenvalues resulting in six principal components, *z*_1_, *z*_2_, *z*_3_, *z*_4_, *z*_5_, and *z*_6_.

It can be seen from Table 2 that for the statistical parameter $x_{3}$, only the principal components $z_{2}$ and $z_{5}$ have positive correlations with it; for the statistical parameter $x_{10}$, the principal components $z_{1}$, $z_{3}$, $z_{4}$, $z_{5}$ and $z_{6}$ have positive correlations with it, and at the same time, $z_{1}$, $z_{3}$, and $z_{5}$ all show strong positive correlations with it. After a comprehensive analysis of the 14 statistical parameters, 3 variables have a positive correlation with at least 4 principal components and at least 3 strong positive correlations, which are as follows: $x_{4}$, $x_{10}$, $x_{11}$. In the same way, variables with a strong positive correlation with the principal components in other PD patterns are obtained, as shown in Table 3.

Based on the above analysis, it can be concluded that for the 14 statistical variables, the seven statistical parameters selected that can better characterize the three PD patterns are: $x_{1}$, $x_{3}$, $x_{4}$, $x_{9}$, $x_{10}$, $x_{11}$, $x_{12}$. The statistical parameters obtained by the above Principal Component Analysis can effectively characterize different types of single PD patterns, which will be used as the input data of the BPNN for training.

## 3. Mantis Search Algorithm and Its Improvements

### 3.1. Mantis Search Algorithm

The MSA is a global optimization algorithm inspired by the natural behavior of praying mantises. Praying mantises are capable of capturing prey through camouflage or confrontation. They use camouflage to approach their prey closely and then launch a sudden attack using their highly modified forelegs. When searching for food, mantises can detect prey, evade it, and use their forelegs for targeted attacks. Additionally, in the mantis species, females sometimes cannibalize males in the mating process. Inspired by the unique hunting behavior and cannibalistic nature of mantises, the MSA consists of four phases: initialization, searching for prey (exploration), attacking prey (exploitation), and sexual cannibalism. Equations and definitions for updating the mantis positions in each phase are listed in Table 4. Detailed explanations can be found in [30]. The MSA boasts several advantages: it is easy to implement, preserves the population diversity in optimization, demonstrates a high ability to escape local optima, possesses a strong exploitation operator for solving unimodal functions, and effectively balances between exploration and exploitation searches. The algorithm’s performance is rigorously evaluated across fifty-two optimization problems and three real-world applications, including five engineering design problems, as well as parameter estimation problems related to photovoltaic modules and fuel cells [30]. These tests demonstrate the algorithm’s versatility and adaptability across diverse scenarios. 

The main parameters of the MSA include the maximum number of iterations, *T*; the population size, *N;* the probability of an attack failure, a; the probability of exchange between the exploration and the exploitation phases, p; the probability for the determining sexual phase, $P_{c}$; etc. Compared to other heuristic algorithms, MSA is simple to implement and has a sufficient population diversity and a strong global searching ability. However, in practical applications, MSA requires an accurate and deliberate adjustment of the control parameters to maximize its performance. Nevertheless, some parameters, such as parameters p and $P_{c}$, are kept fixed in the existing algorithms. Furthermore, it is found that the position updating in the MSA algorithm can easily leap out of the search space. To solve this issue, a re-initialization mechanism, that is, to generate a new position through Equation (5) to replace the position that leapt out of the search space after updating, is introduced in the MSA of [30]. However, this will result in a high computational cost and a low search efficiency. In response to the above problems, this paper proposes some improvements to the MSA in parameter tuning and out-of-bounds updating control.

### 3.2. Improvements on Mantis Search Algorithm

As mentioned above, to address the deficiencies of using fixed control parameters and a lack of search focus in the evolution process of the MSA, this paper proposes adaptive handling mechanisms of the probability p for exchanging between exploration and exploitation phases and the probability $P_{c}$ for determining whether to enter the sexual cannibalism phase. Furthermore, to address the point of easily leaping out of the search space in each step of the original MSA, this paper applies a boundary handling approach to the positions exceeding the feasible space.

#### 3.2.1. Adaptive Updating of Algorithm Parameters

In the MSA, the probability parameter p is used to balance the exploration and the exploitation searches. At the early stage of evolution, p should be large enough to guarantee the diversity of the algorithm while at the late stage of evolution, p should be small enough to bias exploitation searches to speed up the convergence of the algorithm. In this point of view, parameter p is proposed to be adaptively updated by using the following:

(14) $$p=\frac{1+e^{-10\frac{t}{T}}}{2}$$

where t is the generation index of evolution. Obviously, at the early stage of evolution, p is set to be 1, which is conducive to exploration; at the late stage of evolution p is set to be 0.5 to ensure that the exploration and the exploitation searches have the same probability.

In nature, a mantis will become sexually mature incrementally. In line with this natural law, the probability parameter $P_{c}$ should also increase incrementally. In this regard, the probability $P_{c}$ of entering the sexual cannibalism phase is adaptively updated by using the following:

(15) $$P_{c}=\frac{P_{c0}}{1+e^{-10\frac{t}{T}}}$$

where $P_{c0}$ is a coefficient determined by users. This paper sets it to be 0.2 as in the original MSA. Obviously, this probability during the early stage of evolution is approximately 0.16, while it approaches 0.3 during the later stage. The smaller probability in the early stage aligns with the fact that the mantis population is becoming mature, and the mating probability between male and female mantises is increasing, which is more in line with natural laws.

**Figure 4:**
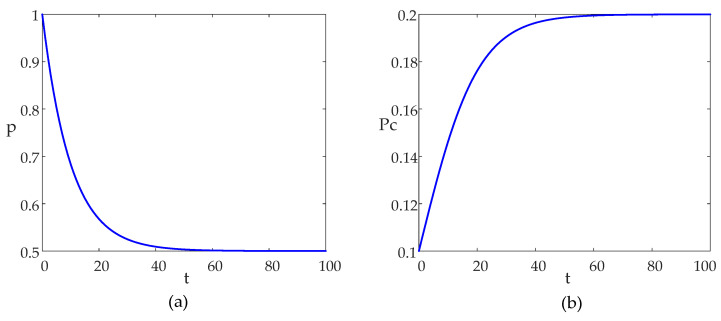
Adaptive updating curves of (**a**) parameter *p*, (**b**) parameter *P_c_*.

The adaptive updating curves of p and $P_{c}$ are shown in Figure 4 under T being 100.

#### 3.2.2. Out-of-Bounds Control

Since the existing strategies of updating the mantises’ position based on hunting behaviors and sexual cannibalism characteristics are prone to escape the search space during the optimization, the improved MSA proposes some boundary handling techniques to the position updating for Equations (6)–(13) of Table 4, replacing the original ones used in MSA. By ensuring that the position updating remains within the feasible space while retaining the original movement information, the algorithm’s optimization efficiency is significantly enhanced.

To ease the boundary handling control explanation, one firstly quantitatively analyses the variation rang of the position variable in the equation in question. Taking Equation (6) as an example, for the *j*th variable of the *i*th mantis ${\vec{x}}_{i}$, when $r_{1}\leq r_{2}$, its value is between $\left[3x_{j}^{l}-2x_{j}^{u},\;3x_{j}^{u}-2x_{j}^{l}\right]$; when $r_{1}>r_{2}$, its value is between $\left[2x_{j}^{l}-x_{j}^{u},2x_{j}^{u}-x_{j}^{l}\right]$, where $r_{1}$ and $r_{2}$ are two random numbers between [0, 1], $x_{j}^{l}$ is the lower bound of the value of the *j*th variable $x_{j}$, $x_{j}^{u}$ is the upper bound of the value of $x_{j}$. From the above analysis, Equation (6) will easily jump out of its value range, $\left[x_{j}^{l},x_{j}^{u}\right]$, after mantis position updating. Therefore, after the position updating by using Equation (6), one first determines whether the updated position, ${\vec{x}}_{i}^{t+1}$, are out of bounds. For the case that the updated *j*th variable jumps out of the feasible space, one then combines it with its variation range using Equation (16), that is, normalizes the variables using the current variation range into the interval $\left[x_{j}^{l},x_{j}^{u}\right]$, and the obtained ${x_{i}^{t+1}}^{\prime }$ is the new position of the mantis. Moreover,

(16) $${x_{i,j}^{t+1}}^{\prime }=\{\begin{array}{ll} x_{j}^{l}+\frac{x_{i,j}^{t+1}+2x_{j}^{u}-3x_{j}^{l}}{5x_{j}^{u}-5x_{j}^{l}}, & r_{1}\leq r_{2}\\ x_{j}^{l}+\frac{x_{i,j}^{t+1}+x_{j}^{u}-2x_{j}^{l}}{3x_{j}^{u}-3x_{j}^{l}}, & \mathrm{Otherwise}\; \end{array}$$

The (position) variable variation ranges of Equations (6)–(13) are shown in Table 5. When a mantis position jumps out of the search space, it is processed according to these ranges, $\left[x_{j}^{\min},x_{j}^{\max}\right]$, of its equation, so that it is forced to return to the feasible space, as shown in Equation (17), where $x_{j}^{\min}$ is the minimum value of the variable in the equation in question, and $x_{j}^{\max}$ is the maximum value of the variable in the equation in question.

(17) $${x_{i,j}^{t+1}}^{\prime }=x_{j}^{l}+\frac{x_{i,j}^{t+1}-x_{j}^{\min}}{x_{j}^{\max}-x_{j}^{\min}}$$

#### 3.2.3. Flowchart of Improved Mantis Search Algorithm

**Figure 5:**
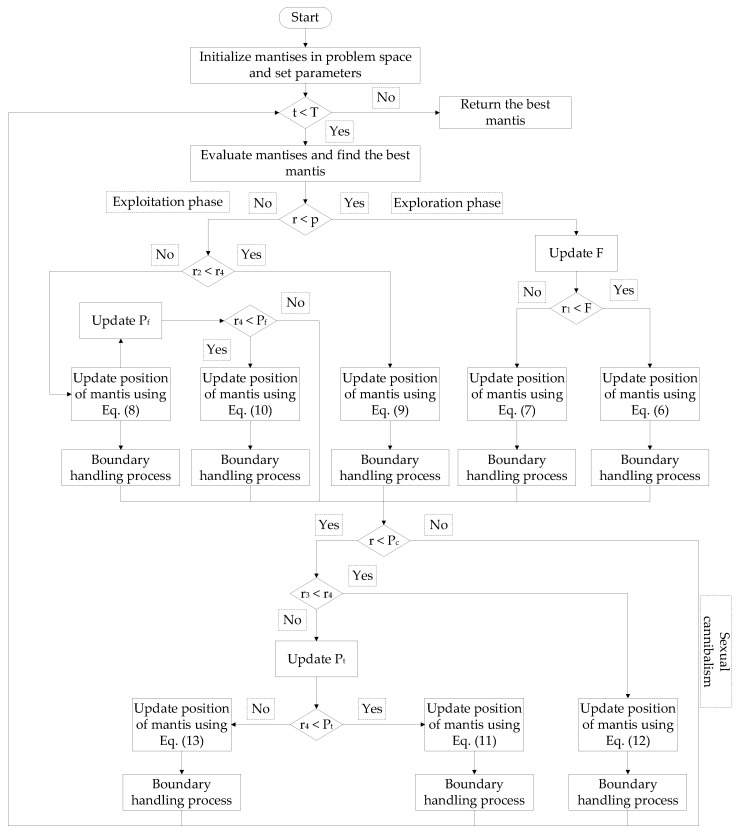
Improved MSA flowchart.

To facilitate the implementation of the improved MSA, its flowchart is shown in Figure 5.

## 4. PDPR of Switchgear Based on Improved Mantis Search Algorithm—Back Propagation Neural Network

### 4.1. PDPR Process and Parameter Settings

**Figure 6:**
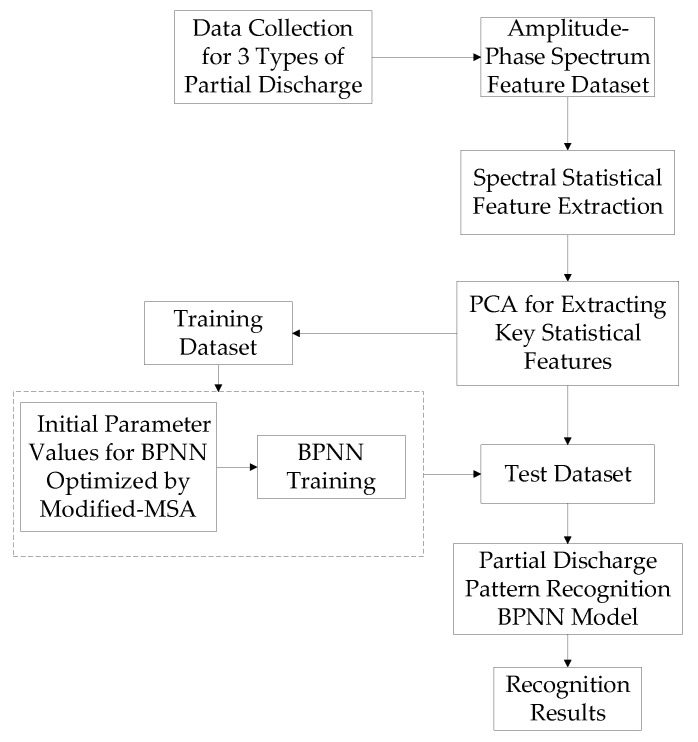
PDPR process based on improved MSA-BPNN.

As explained previously, a BPNN is a multi-layer feedforward network with strong nonlinear approximation capabilities. Existing research has demonstrated the effectiveness of BPNNs in PDPR [13,28]. However, the BPNN classification method exhibits some subjectivity in feature selections, often relying on expert experience, resulting in a significant information loss and a lack of generalization, leading to low recognition rates [28]. To address these deficiencies, this paper employs PCA to reduce the dimensionality of the features of the discharge data obtained from ultrasonic detections. The processed features are then input into BPNN for learning, enabling the recognition of partial discharge patterns. Additionally, the BPNN is sensitive to the choice of initial values of network parameters, and the algorithm requires a long training time and is prone to getting stuck in local minima [29]. To overcome the drawback of BPNN potentially converging to local optima, this paper utilizes the improved MSA to conduct a global search for the initial parameters of BPNN. The complete PDPR process is shown in Figure 6.

**Figure 7:**
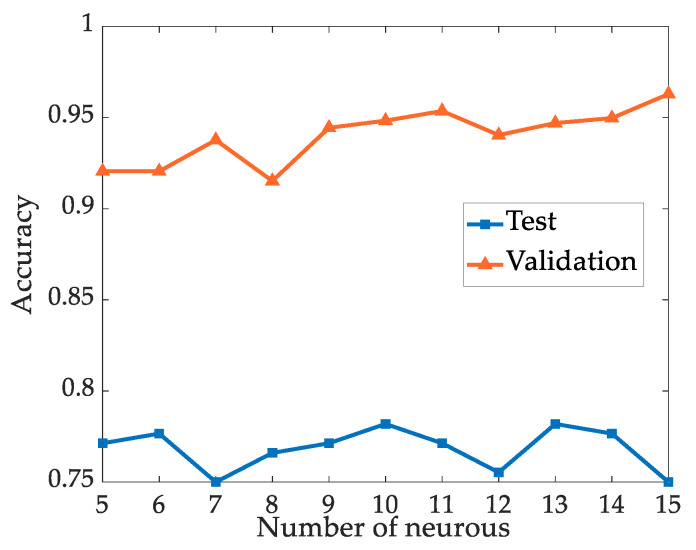
Recognition accuracy with different numbers of neurons in the hidden layer.

The node number of the input layer of the BP neural network is equal to the number of statistical feature parameters, and the output layer has three neurons representing three types of discharge patterns [8], i.e., [1 0 0] represents metal particle discharge, [0 1 0] represents suspended discharge, and [0 0 1] represents creeping discharge. Initially, the number of neurons in the hidden layer is initially fixed at 10 according to [34], and experiments are conducted using a data set with 14 statistical features. The number of neurons varies from 5 to 15, and the recognition accuracy is shown in Figure 7. From Figure 7, it can be observed that within the range of experimental neuron numbers, the recognition accuracy does not exhibit a linear proportional relationship with the number of neurons. Specifically, the validation accuracy peaks with 15 neurons, whereas the test accuracy achieves its peak with 10 and 13 neurons. Furthermore, as the number of neurons increases, the computational load of the network inevitably rises. Considering both accuracy and computational load, the number of neurons in the hidden layer of this study is set at 10. 

Ultrasonic testing is used for measurements, where four-fifths of each discharge mode’s data are used for training and one-fifth for testing. In the study, there are 388 training data sets and 97 testing data sets for metal particle discharge, 318 training data sets and 79 testing data sets for suspended discharge, 49 training data sets, and 12 testing data sets for creeping discharge.

The objective function is to minimize the root mean square error (RMSE) of the BPNN:

(18) $$\min{\;f}_{objective}=\sqrt{\frac{\sum_{i=1}^{I}{\left(y_{out}-y_{test}\right)}^{2}}{I}}$$

where $y_{out}$ is the measurement output, $y_{test}$ is the neural network output, I is the number of data sets.

### 4.2. Experimental Results

**Figure 8:**
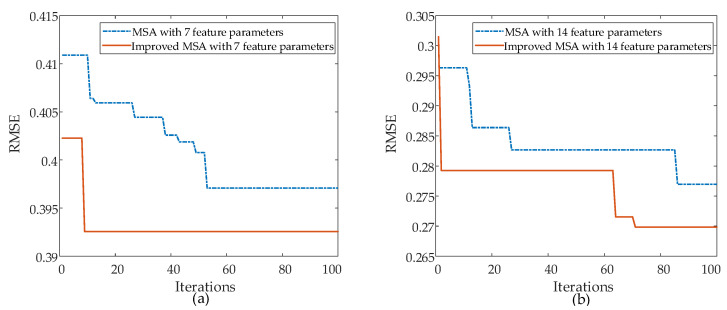
Comparison of MSA and Improved MSA algorithms with (**a**) 7 feature parameters, (**b**) 14 feature parameters.

The optimized results of BPNN parameters using different approaches, including using MSA optimization algorithm and using the proposed improved MSA optimization algorithm, are compared. To further compare the effectiveness of the selected seven features by PCA, the study also compared the training of the BPNN model for partial discharge pattern recognition using the original 14 features. The experiments were conducted using MATLAB R2022b on a system equipped with an AMD Ryzen 7 4800H processor featuring Radeon Graphics operating at 2.90 GHz, coupled with 16.0 GB of RAM. The algorithm parameters for MSA and the improved MSA algorithm are shown in Table 6, and the obtained results are shown in Figure 8. From Figure 8, it can be observed that after 100 iterations of optimizations, the BPNN optimized using the improved MSA gives smaller errors in both the 7 extracted statistical feature parameters and 14 statistical feature parameters. Furthermore, the improved MSA algorithm exhibits a faster convergence and a higher efficiency as compared to the original MSA in optimizing the BPNN.

In the final determination of partial discharge types, the maximum output value will be set to 1, and the other values will be set to 0, corresponding to the three discharge modes. The accuracies of the training data and testing data using different classifiers are shown in Table 7. To demonstrate the efficiency of PCA, the results of testing the training data and testing data using different classifiers trained by all 14 extracted statistical feature parameters are shown in Table 8.

In the PDPR results of the BPNN trained with seven features, both for training and testing data, the recognition accuracy of the BPNN optimized by the improved MSA is higher than that of the BPNN optimized by the original MSA. The same conclusion is drawn from the PDPR results of the BPNN trained with 14 features, as shown in Table 8. Comparing the BPNN training using 7 and 14 features, the accuracy using 14 features is higher on the training data; however, on the testing data set, the recognition efficiency of the two is comparable. Taking the example of using the improved MSA-BPNN, the recognition accuracy for metal particle discharge using 7 features is 92.59%, which is lower than the corresponding model using 14 features. However, the model trained with 7 features for suspended discharge recognition is 64.39%, higher than the 62.88% achieved with 14 features. For surface discharge, both have an equal accuracy of 90% in testing data. Furthermore, when using 7 features, the BPNN has 113 network parameters, while using 14 features, the BPNN has 183 network parameters. From the runtime data in Table 7 and Table 8, it is evident that using a comprehensive but minimal number of features can reduce runtime while ensuring the accuracy of PDPRs.

**Figure 9:**
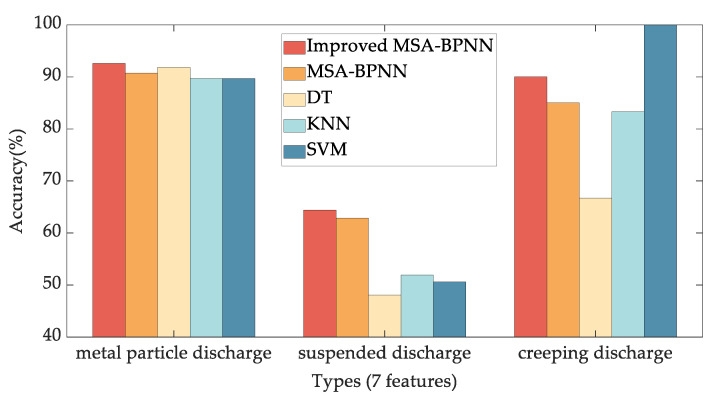
Comparison of test accuracy results of Improved MSA-BPNN, MSA-BPNN, DT, KNN, and SVM, using 7 statistical feature parameters.

**Figure 10:**
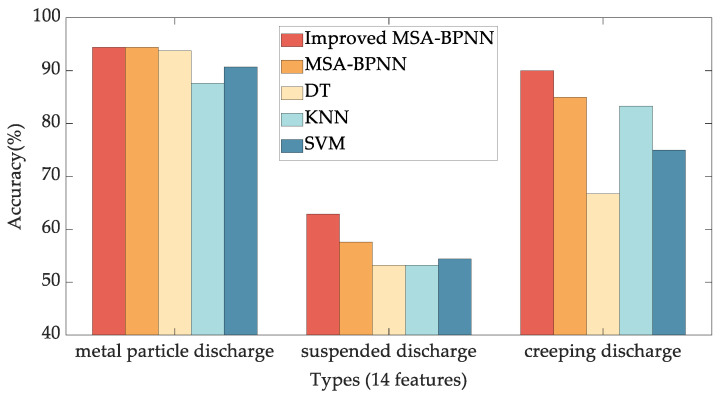
Comparison of test accuracy results of Improved MSA-BPNN, MSA-BPNN, DT, KNN, and SVM, using 14 statistical feature parameters.

To assess the merits of the proposed method, this paper also compares the pattern recognition accuracy rates of Decision Tree (DT) [35], k-Nearest Neighbor classifiers (KNN) [36], and Support Vector Machine (SVM) [37,38]. Experiments using fine DT, fine KNN, and cubic SVM are conducted on the Classification Learner App in MATLAB R2022b. The test accuracy results using 7 features and 14 features are shown in Figure 9 and Figure 10, respectively.

From Figure 9 and Figure 10, it is evident that the Improved MSA-BPNN proposed in this paper exhibits the highest overall recognition accuracy. Compared to using the 7 features after PCA, the recognition accuracy using the original 14 features is slightly higher. Specifically, in the case of the suspended discharge with 7 features, the recognition accuracies for Improved MSA-BPNN, MSA-BPNN, DT, KNN, and SVM are 64.39%, 62.88%, 48.1%, 51.9%, and 50.6%, respectively, while with 14 features, the recognition accuracies are 62.88%, 57.58%, 53.2%, 53.2%, and 54.4%. Moreover, the SVM achieves 100% recognition accuracy for creeping discharge when utilizing 7 features. However, with 14 features, the recognition accuracy decreases to 75%. This decline can be attributed in part to the relatively low number of samples associated with creeping discharge, which is the least represented among the three types of discharges. Moreover, this outcome underscores the effectiveness of PCA in reducing the number of features while still maintaining effective recognition accuracy.

It should be pointed out that, the most damage to low- and medium-voltage switchboards comes from the overloads, and the temperature control itself allows us to predict a possible failure in advance. Nevertheless, in this paper, we have focused on using ultrasonic signals to identify types of partial discharge faults in a switchgear. The presented algorithm is still valid if one can abstract the features of the local over heat and use these features as the input of the subsequent procedure of the proposed algorithm.

## 5. Conclusions

The reliable operation of the switchgear is crucial to the reliability of the power supply of the power system. From this point of view, it is of great significance to perform PDPR on the switchgear. BPNNs are widely used for PDPR. However, the BPNN has its inherent limitations and shortcomings, such as requiring a long training time and converging to local minimums, etc., making it incompetent in engineering applications.

This paper uses PCA to reduce the dimensionality of statistical feature parameters obtained from PRPD spectrograms. It extracts the main features as the inputs of the BPNN for training to recognize the partial discharge patterns. To address the high sensitivity of BPNNs to the initial values of the network parameters, this paper adopts an improved MSA to conduct global searches for the parameters of the BPNN and determine the initial values of the BPNN, thereby reducing the training time and overcoming the drawback of potentially converging to local optima in BPNN. The improved MSA handles boundary conditions in each step of the search when the position is moved out of the search space. By retaining the original movement information, the search is forced back into the feasible space, greatly improving the solution efficiency of the algorithm. Experimental results show that compared to the MSA, the BPNN optimized by the improved MSA has smaller errors. Compared to the utilization of 14 feature parameters, the application of seven features derived through PCA achieves a commensurate recognition accuracy in PDPRs, evidently resulting in time savings. The results of the algorithm are also compared with three commonly used algorithms, DT, KNN, and SVM, in PDPRs. The recognition accuracy results demonstrate the effectiveness of the algorithm proposed in this paper.

In engineering applications, the presented algorithm can be used in a system to monitor if a distribution device has experienced a partial discharge fault and to identify the types of a partial discharge fault (if yes) from the measured ultrasonic signals.

In this study, the primary emphasis is on signals obtained by ultrasonic detection methods, without conducting an experimental analysis of signals acquired from other types of sensors. Our future endeavors will involve applying the algorithm developed in this study to recognize partial discharge patterns using different types of signals to augment the precision of pattern recognitions.

## Figures and Tables

**Table 1:** Fourteen statistical parameters.

Statistical Parameters	$H_{u\max}(\varphi )$		$H_{u\mathrm{mean}}(\varphi )$	
	+	−	+	−
Sk	$x_{1}$($Sk_{\max}^{+}$)	$x_{2}$($Sk_{\max}^{-}$)	$x_{3}$($Sk_{mean}^{+}$)	$x_{4}$($Sk_{mean}^{-}$)
Ku	$x_{5}$($Ku_{\max}^{+}$)	$x_{6}$($Ku_{\max}^{-}$)	$x_{7}$($Ku_{mean}^{+}$)	$x_{8}$($Ku_{mean}^{-}$)
Pe	$x_{9}$($Pe_{\max}^{+}$)	$x_{10}$($Pe_{\max}^{-}$)	$x_{11}$($Pe_{mean}^{+}$)	$x_{12}$($Pe_{mean}^{-}$)
cc	$x_{13}$($cc_{\max}$)		$x_{14}$($cc_{mean}$)	

**Table 2:** Principal coefficients of statistical parameters of suspended discharge.

Statistical Parameters	Principal Component Coefficients					
	*z*_1_	*z*_2_	*z*_3_	*z*_4_	*z*_5_	*z*_6_
*x*_1_	−0.070	0.203	−0.129	−0.204	0.060	0.030
*x*_2_	0.126	−0.396	0.159	0.349	−0.100	−0.396
*x*_3_	−0.080	0.173	−0.118	−0.107	0.083	−0.405
***x*_4_**	**0.170**	−0.284	**0.116**	**0.146**	−0.126	**0.577**
*x*_5_	−0.021	0.048	−0.008	−0.035	0.013	0.075
*x*_6_	−0.126	0.383	−0.129	−0.271	0.079	0.409
*x*_7_	−0.031	0.046	−0.026	−0.032	0.025	−0.104
*x*_8_	−0.141	0.221	−0.080	−0.105	0.084	−0.377
*x*_9_	0.166	0.363	0.872	−0.180	−0.202	−0.067
***x*_10_**	**0.310**	−0.056	**0.188**	**0.024**	**0.929**	**0.033**
***x*_11_**	**0.405**	**0.590**	−0.202	**0.663**	−0.076	**0.025**
*x*_12_	0.787	−0.093	−0.265	−0.494	−0.198	−0.116
*x*_13_	0.002	−0.008	0.004	0.008	−0.002	−0.008
*x*_14_	0.004	−0.006	0.004	0.004	−0.003	0.012

**Table 3:** Variables with strong positive correlation with principal components under different PD patterns.

Detection Approach	Partial Discharge Mode		
	Metal Particle Discharge	Suspended Discharge	Creeping Discharge
Ultrasonic testing	$x_{9}$, $x_{12}$	$x_{4}$, $x_{10}$, $x_{11}$	$x_{1}$, $x_{3}$, $x_{11}$, $x_{12}$

**Table 4:** Position updating equation of MSA.

Phase	Equations	Explanations	Number
Initialization	${\vec{x}}_{i}^{t}={\vec{x}}^{l}+\vec{r}*\left({\vec{x}}^{u}-{\vec{x}}^{l}\right)$	Generate initial positions of the population.	(5)
Exploration	${\vec{x}}_{i}^{t+1}=\{\begin{array}{ll} {\vec{x}}_{i}^{t}+{\vec{\tau }}_{1}*\left({\vec{x}}_{i}^{t}-{\vec{x}}_{a}^{t}\right)+\left|\tau_{2}\right|\cdot \vec{U}*\left({\vec{x}}_{a}^{t}-{\vec{x}}_{b}^{t}\right), & r_{1}\leq r_{2}\\ {\vec{x}}_{i}^{t}*\vec{U}+\left({\vec{x}}_{a}^{t}+{\vec{r}}_{3}*\left({\vec{x}}_{b}^{t}-{\vec{x}}_{c}^{t}\right)\right)*(1-\vec{U}), & \;\mathrm{Otherwise}\; \end{array}$	Mantises usually stay motionless on branches or in weeds, waiting for prey to approach an ambush distance before striking.	(6)
	${\vec{x}}_{i}^{t+1}=\{\begin{array}{ll} {\vec{x}}_{i}^{t}+\alpha \cdot \left({\vec{x}}_{ar}^{\prime }-{\vec{x}}_{a}^{t}\right), & r_{9}\leq r_{10}\\ {\vec{x}}_{ar}^{\prime }+\left(r_{7}*2-1\right)*\mu *\left({\vec{x}}^{l}+{\vec{r}}_{8}\times \left({\vec{x}}^{u}-{\vec{x}}^{l}\right)\right), & \mathrm{Otherwise}\; \end{array}$	Find prey without using camouflage.	(7)
Exploitation	$x_{i,j}^{t+1}=\left(x_{i,j}^{t}+x_{j}^{*}\right)/2.0+v_{s}\cdot d_{si,j}^{t}$	Mantis uses the front legs to attack prey.	(8)
	$x_{i,j}^{t+1}=x_{i,j}^{t}+r_{12}\cdot \left(x_{a,j}^{t}-x_{b,j}^{t}\right)$	If the mantis fails to attack, it needs to change direction before attacking again.	(9)
	$x_{i,j}^{t+1}=x_{i,j}^{t}+e^{2l}\cdot \cos(2l\pi )\cdot \left|x_{i,j}^{t}-{\vec{x}}_{ar,j}^{\prime }\right|+\left(r_{13}\cdot 2-1\right)\cdot \left(x_{j}^{u}-x_{j}^{l}\right)$	Failure of the mantis attack means that the mantis fell into the trap of the local optimal solution. Mantis should escape from the local optimal solution.	(10)
Sexual cannibalism	${\vec{x}}_{i}^{t+1}={\vec{x}}_{i}^{t}+{\vec{r}}_{16}*\left({\vec{x}}_{i}^{t}-{\vec{x}}_{a}^{t}\right)$	Female mantises attract male mantises to their location by attracting them.	(11)
	${\vec{x}}_{i}^{t+1}={\vec{x}}_{i}^{t}*\vec{U}+\left(x_{11}^{t}+{\vec{r}}_{18}*\left(-x_{11}^{t}+{\vec{x}}_{i}^{t}\right)\right)*(1-\vec{U})$	A male mate and a female to produce a new offspring.	(12)
	${\vec{x}}_{i}^{t+1}={\vec{x}}_{a}^{t}\cdot \cos(2\pi l)\cdot \mu$	Female eats male.	(13)

**Table 5:** Updated position range.

Phase	Equation Number	Variable Variation Ranges
Exploration	(6)	$\begin{array}{l} {}[3x_{j}^{l}-2x_{j}^{u},\;3x_{j}^{u}-2x_{j}^{l}],\;r_{1}\leq r_{2}\\ {}[2x_{j}^{l}-x_{j}^{u},2x_{j}^{u}-x_{j}^{l}],\;\mathrm{Otherwise} \end{array}$
	(7)	$\begin{array}{l} {}[2x_{j}^{l}-x_{j}^{u},\;2x_{j}^{u}-x_{j}^{l}],r_{9}\leq r_{10}\\ {}[x_{j}^{l}-x_{j}^{u},\;2x_{j}^{u}],\;\mathrm{Otherwise}\; \end{array}$
Exploitation	(8)	$\left[-\frac{1}{2}x_{j}^{l},\frac{3}{2}x_{j}^{u}\right]$
	(9)	$\left[2x_{j}^{l}-x_{j}^{u},2x_{j}^{u}-x_{j}^{l}\right]$
	(10)	$\left[x_{j}^{l}+(-e^{-3}-1)(x_{j}^{u}-x_{j}^{l}),\;x_{j}^{u}+(e^{-2}+1)(x_{j}^{u}-x_{j}^{l})\right]$
Sexual cannibalism	(11)	$\left[2x_{j}^{l}-x_{j}^{u},\;2x_{j}^{u}-x_{j}^{l}\right]$
	(12)	$\left[x_{j}^{l},\;x_{j}^{u}\right]$
	(13)	$\left[-x_{j}^{l},\;x_{j}^{u}\right]$

**Table 6:** MSA and improved MSA algorithm parameter settings.

Parameter	MSA	Improved MSA
N	30	30
T	100	100
p	0.5	$p=\frac{1+e^{-10\frac{t}{T}}}{2}$
a	0.5	0.5
$P_{c}$	0.2	$P_{c}=\frac{P_{c0}}{1+e^{-10\frac{t}{T}}}$

**Table 7:** PD pattern recognition accuracies using 7 statistical feature parameters.

Classifier Model	Discharge Pattern	Validation Accuracy	Test Accuracy	Runtime
Improved MSA-BPNN	metal particle discharge	91.33%	92.59%	427.4 s
	suspended discharge	89.81%	64.39%	
	creeping discharge	95.12%	90.00%	
MSA-BPNN	metal particle discharge	90.09%	90.74%	379.6 s
	suspended discharge	89.06%	62.88%	
	creeping discharge	92.68%	85.00%	

**Table 8:** PD pattern recognition accuracies using 14 statistical feature parameters.

Classifier Model	Discharge Pattern	Validation Accuracy	Test Accuracy	Runtime
Improved MSA-BPNN	metal particle discharge	98.45%	94.44%	697.7 s
	suspended discharge	97.73%	62.88%	
	creeping discharge	100.0%	90%	
MSA-BPNN	metal particle discharge	97.83%	94.44%	610.6 s
	suspended discharge	97.36%	57.58%	
	creeping discharge	100.0%	85.00%	

## Data Availability

The original contributions presented in the study are included in the article, further inquiries can be directed to the corresponding author.
